# Identification of an Unpredicted GAG-PUL in *Roseihalotalea indica* gen. nov. sp. nov. TK19036^T^ and Characterization of Novel GAG-Lyases with Unique Substrate Specificities

**DOI:** 10.3390/md24030115

**Published:** 2026-03-20

**Authors:** Zheng Fu, Defang Wu, Shunqin You, Kai Tang, Runying Zeng, Zhuhua Chan

**Affiliations:** 1 Technology Innovation Center for Exploitation of Marine Biological Resources, Third Institute of Oceanography, Ministry of Natural Resources, Xiamen 361005, China; fuzheng@tio.org.cn; 2 School of Life Sciences and Biopharmaceutics, Shenyang Pharmaceutical University, Shenyang 117004, China; 3 State Key Laboratory of Marine Environmental Science, College of Ocean and Earth Science, Fujian Key Laboratory of Marine Carbon Sequestration, Xiamen University, Xiamen 361005, China

**Keywords:** *Roseihalotalea indica*, glycosaminoglycans, glycosaminoglycan lyase, DIA quantitative proteomics, polysaccharide utilization locus

## Abstract

Glycosaminoglycans (GAGs) and their degrading enzymes have extensive applications and biotechnology and medicine, and play a crucial role in the recycling of organic matter in oceans. In this study, a potential GAG utilization gene cluster was identified in the genome of a novel marine *Bacteroidetes*, *Roseihalotalea indica* gen. nov. sp. nov. TK19036^T^, through sole carbon source cultivation and differential proteomic analysis. Multiple GAG-lyases within this locus were purified and characterized. RiPL8 comprises a functionally unknown N-terminal domain and a catalytic C-terminal domain, exhibiting specificity for degrading hyaluronic acid (HA). The activity of RiPL35 is sensitive to Ca^2+^ ion concentration with an optimum at 10 mM. RiPL38 is the first reported member of the PL38 family capable of degrading HA and chondroitin sulfate (CS). In summary, our study reveals *Roseihalotalea indica* gen. nov. sp. nov. TK19036^T^ harbors an unpredicted GAG degradation gene cluster, and the encoded GAG-lyases exhibit distinct substrate specificities compared to the host organism.

## 1. Introduction

**Figure 1:**
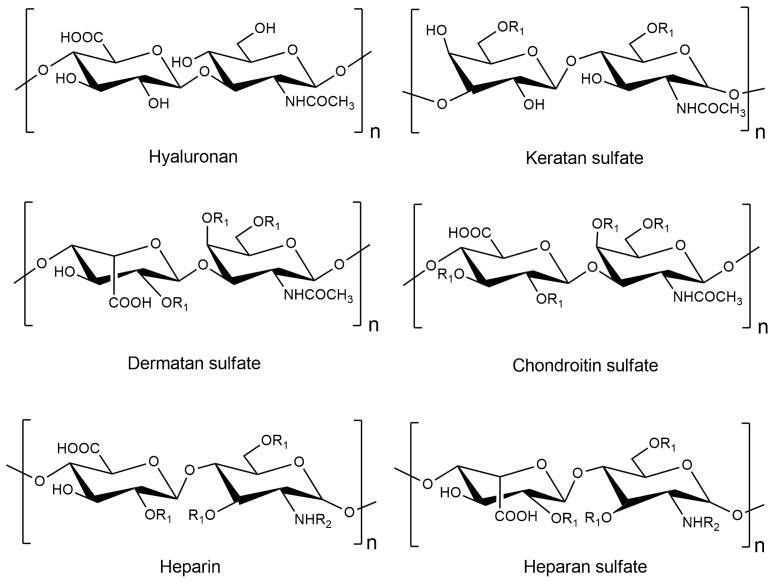
Major disaccharide repeating units of different types of GAGs. R refers to the substituent labeled R_1_ = H/SO^3−^ and R_2_ = H/SO^3−^/COCH^3−^.

Glycosaminoglycans (GAGs) are structurally diverse linear heteropolysaccharides that serve as the carbohydrate moieties of proteoglycans and are ubiquitously localized in the extracellular matrix and cell membranes of all tissues. They consist of repeating disaccharide units (hexosamine and hexuronic acid) linked via β-1,4 and β-1,3 glycosidic bonds [1]. Based on disaccharide unit structure, GAG is mainly classified into the following categories: hyaluronan (HA), keratan sulfate (KS), chondroitin sulfate (CS), dermatan sulfate (DS), heparin, and heparan sulfate (HS) (Figure 1). Interactions between GAGs with proteins mediate diverse biological processes, including cell adhesion, growth, differentiation, signaling, wound healing, smooth articulation, host defense, viral infection, vascular generation, blood coagulation, inflammation, and cancer [1]. As important marine polysaccharides, GAGs are extracted from marine animals such as invertebrates, mollusks, sea cucumbers, sponges, and squids, primarily from cartilaginous material of fish (shark, salmon, ray, etc.), and are widely used in food, cosmetic, and clinical industries [2].

GAG-degrading enzyme, a type of enzyme that degrades GAG by specific hydrolysis or cleavage of β-1,3 glycosidic or β-1,4 glycosidic bonds, can be further divided into glycoside hydrolase (GH) and polysaccharide lyase (PL) [3]. Of these, GAG-lyase, derived only from microorganisms, can catalyze the cleavage of 1,4-glycoside bonds via a β-elimination mechanism, producing GAG unsaturated oligosaccharides, which possess unsaturated bonds at the non-reducing end [4]. According to the optimum substrate, it can be classified into three categories based on the optimum substrate, hyaluronate lyase, chondroitin sulfate lyase, and heparin lyase. Some GAG-lyases have the limited ability to degrade other GAGs, albeit at a slower rate. GAG-lyase can be divided into 14 PL families, including PL-6, -8, -12, -13, -15, -16, -17, -21, -23, -29, -30, -33, -35, and -37 based on amino acid sequence similarities [5].

In recent decades, a diverse array of bacterial GAG-lyases has been identified from various microorganisms, including *Bacteroides* sp. [6], *Clostridium* sp. [7], *Enterococcus* sp. [8], *Escherichia* sp. [9], *Hungatella* sp. [10], *Microbacterium* sp. [11], *Segatella* sp. [12], *Yersinia* sp. [13], and *Vibrio* sp. [14]. The degradation of GAG requires synergistic action of multiple proteins, viz. GAG-degrading enzymes (PLs or GHs), esterase, disaccharide hydrolase, sugar transporters, and transcriptional factors. Additionally, sulfatases are necessary for complete degradation to sulfated GAGs [15]. These genes are frequently organized into a polysaccharide utilization locus (PUL), which coordinates the processes of sensing, enzymatic digestion, transport, and metabolism of a specific polysaccharide. In *Bacteroidetes*, PULs are characterized by the presence of homologous pairs of signature genes, notably an outer membrane TonB-dependent transporter (also known as SusC) and a cell surface glycan-binding protein (also known as SusD) [16].

This study aims to investigate the GAG utilization mechanism of the novel marine *Bacteroidetes*, *Roseihalotalea indica* gen. nov. sp. nov. TK19036^T^, previously isolated from the Southwest Indian Ocean and characterized by enhanced carbohydrate metabolic potential [17,18], using sole-carbon-source cultivation and DIA quantitative proteomics. We focus on identifying a potential GAG-PUL from this strain and characterizing the enzymatic properties of three polysaccharide lyases (RiPL8, RiPL35, and RiPL38) to elucidate their substrate specificities, thereby advancing the understanding of functional diversity in marine *Bacteroidetes* PULs.

## 2. Results

### 2.1. Analysis of GAG Utilization in Roseihalotalea indica gen. nov. sp. nov. TK19036^T^

**Figure 2:**
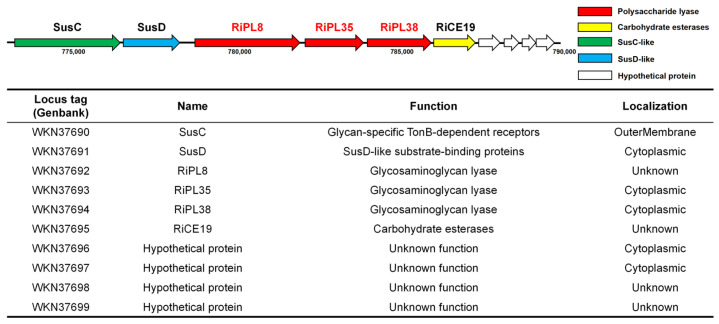
Predicted polysaccharide utilization locus of GAG (GAG-PUL) in *Roseihalotalea indica* gen. nov. sp. nov. TK19036^T^.

*Roseihalotalea indica* gen. nov. sp. nov. TK19036^T^ has been predicted to encode an extensive array of CAZymes and PULs, as previously documented [17,18]. We identified a novel polysaccharide utilization locus between 773,288 bp and 789,641 bp in the genome, which was not predicted by CGC Finder. The cluster contains the gene markers of PUL—SusC and SusD—and four CAZyme genes annotated as in Figure 2, e.g., PL-8 lyase, WKN37692; PL-35 lyase, WKN37693; PL-38 lyase, WKN37694; and CE-19 esterase, WKN37695.

**Figure 3:**
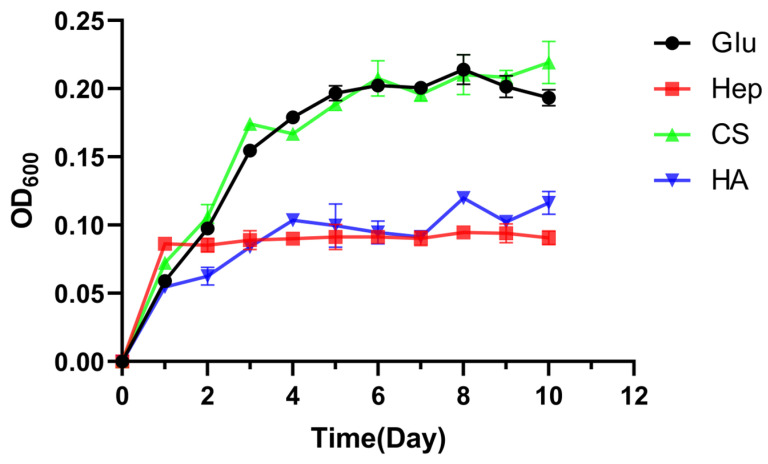
Growth curves of *Roseihalotalea indica* gen. nov. sp. nov. TK19036^T^ in the medium with glucose, heparin, chondroitin sulfate, or hyaluronate as the sole carbon source.

To assess the capacity of *Roseihalotalea indica* gen. nov. sp. nov. TK19036^T^ to metabolize GAGs, a bacterial growth experiment was conducted using HA, CS, heparin, and glucose as the sole carbon sources, with the growth curve monitored as shown in Figure 3. The cells exhibited rapid proliferation in media containing CS or glucose, entering the exponential growth phase and reaching the stationary phase by day 5. Nevertheless, the OD_600_ of the HA and heparin groups remained under 0.1 following inoculation. This result demonstrates that *Roseihalotalea indica* gen. nov. sp. nov. TK19036^T^ could only degrade and utilize CS.

**Figure 4:**
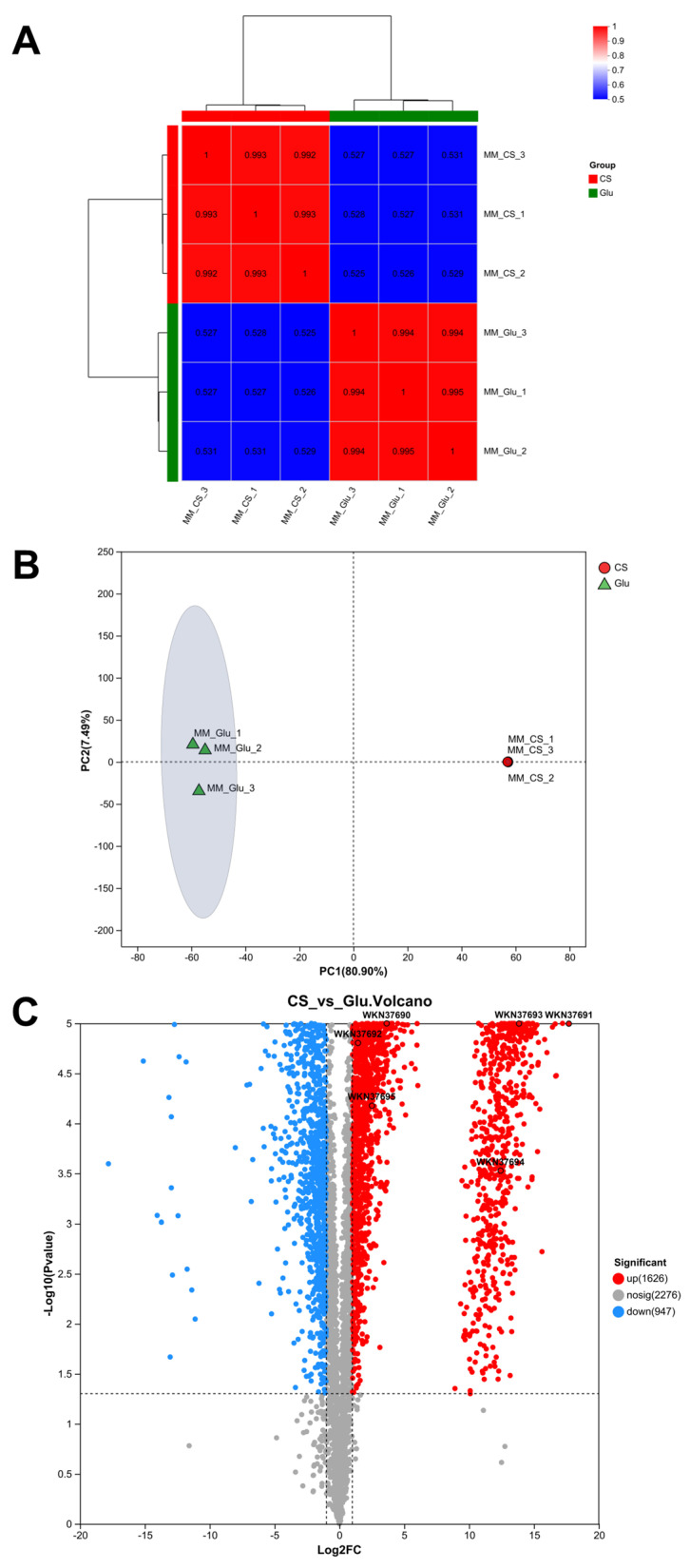
DIA quantitative proteomics of *Roseihalotalea indica* gen. nov. sp. nov. TK19036^T^ under different carbon-source culture (Glu vs. CS). (**A**) Heatmap of the correlation between samples, where the horizontal and vertical coordinates represent the square of the correlation coefficients of each sample. (**B**) PCA plot. The abscissa represents the first principal component, and the ordinate represents the second principal component. (**C**) Volcano plot highlighting differentially expressed proteins, with log2 fold changes against statistical significance (i.e., −log10 *p*-values). Red dots represent upregulated proteins, blue dots show downregulated proteins, and black dots denote proteins with no significant differential expression. Marked points are key proteins in the GAG-PUL.

The data-independent acquisition (DIA) quantitative proteomics method was utilized to examine the CS metabolism of *Roseihalotalea indica* gen. nov. sp. nov. TK19036^T^. We collected samples from the Glu group and the CS group in the previous sole-carbon-source cultivation for proteomics analysis, namely MM_Glu and MM_CS, respectively, and performed three biological replicates, with a total of six samples. A total of 71,750 related peptides and 4997 proteins were identified and retained for comparative analysis. A comparison of the protein intensity correlation coefficients of these samples, where coefficients were >0.5, indicated a high reproducibility, integrity, and reliability of the proteomics data between the Glu and CS (Figure 4A). The heatmap displayed the various relationships of *Roseihalotalea indica* gen. nov. sp. nov. TK19036^T^ under different carbon-source culture conditions.

A subsequent Principal Component Analysis (PCA) confirmed this dramatic difference (Figure 4B). The PCA plot clearly distinguished with confidence ellipses of clusters, indicating that there were significant differences between the Glu and CS groups. The clustering pattern revealed the proteomic profiles of *Roseihalotalea indica* gen. nov. sp. nov. TK19036^T^ under different substrate metabolism conditions, indicating distinct molecular adaptations to utilize CS.

The differentially altered proteins were analyzed using a volcano map (Figure 4C), which displays the relationship between log2 fold changes and significance, represented by −log10 (*p*-value). Red and blue dots represent significantly upregulated (1626) and downregulated (947) proteins, respectively. The upregulated proteins in the GAG-PUL have been labeled in a volcano plot; no significant changes were observed in the expression of hypothetical proteins from WKN37696 to WKN37699. Altogether, these findings suggest that the proteins from WKN37690 to WKN37695 in the GAG-PUL play a key role in the metabolic processing of CS in *Roseihalotalea indica* gen. nov. sp. nov. TK19036^T^.

### 2.2. Sequence Analysis and Recombinant Expression of Key Proteins in GAG-PUL

**Figure 5:**
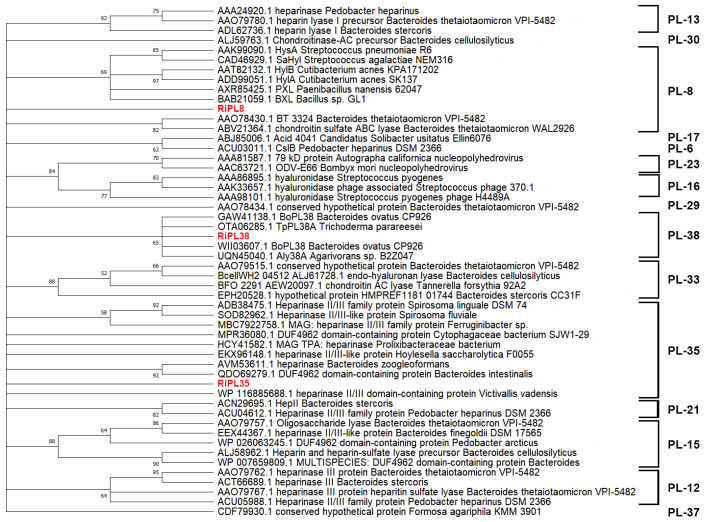
Phylogenetic analysis of RiPL8, RiPL35, RiPL38, and other reported GAG-lyases from different families.

Phylogenetic analysis was performed based on the GAG-lyase sequences from different PL families collected from the CAZy database, illustrating that these three key proteins belong to various PL families (Figure 5).

**Figure 6:**
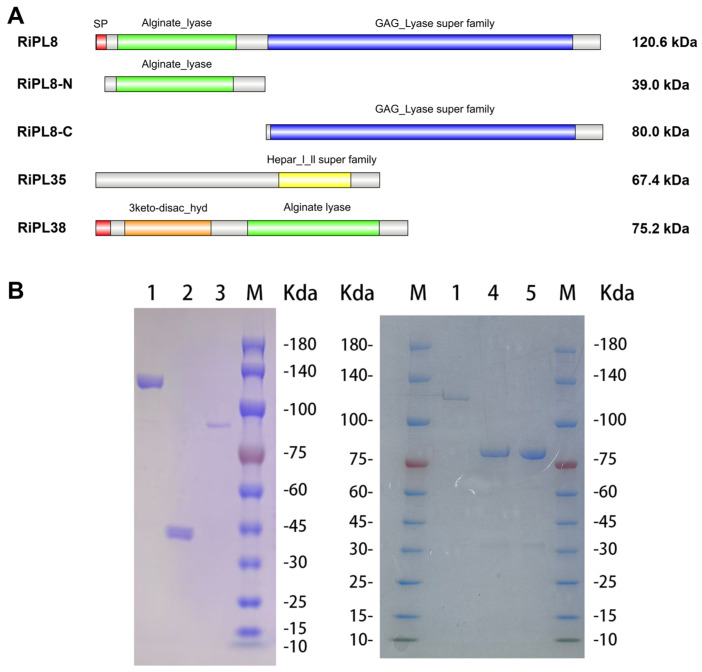
Design and recombinant expression of RiPL35, RiPL38, RiPL8 and its truncated mutant. (**A**) Domain structure of the purified recombinant proteins. (**B**) SDS-PAGE analysis of the purified recombinant proteins. Lane M, molecular weight markers; Lane 1, RiPL8; Lane 2, RiPL8-N; Lane 3, RiPL8-C; Lane 4, RiPL35; Lane 5, RiPL38.

RiPL8 was composed of 1066 amino acids and had an Mw of 120.6 kDa and a pI of 5.24. Additionally, the SignalP 6.0 prediction suggested that RiPL8 contains a signal peptide (Sec/SPI) cleavage site between amino acids 22 and 23. The CD-search analysis showed that RiPL8 contains an “Alginate_lyase” N-terminal domain (Pro^48^-Trp^297^) and a “GAG_Lyase super family” C-terminal module (Glu^365^-Pro^1007^), as illustrated in Figure 6A.

RiPL35 is a protein comprising 600 amino acids, and the theoretical Mw and pI were 67.3 kDa and 4.84, respectively. The signal prediction showed that RiPL35 lacks a signal peptide. The CD-search analysis indicates the presence of a “Hepar_l_ll super family” domain (Ser^388^-Gly^539^) in the RiPL35.

RiPL38 encodes a 660-amino-acid protein with a theoretical Mw of 75.1 kDa and pI of 4.86. Based on SignalP 6.0, RiPL38 contains a 32-residue Sec/SPII signal peptide (Met^1^-Ala^32^), a “3keto-disac_hyd” module (Gln^62^-Ile^244^), and a “Alginate lyase” domain (The^321^-Trp^599^).

RiPL35, RiPL38, RiPL8 and its truncated mutant remove the signal peptide and were purified via a HisTrap HP column after prokaryotic expression in *E. coli* BL21 (DE3) cells. The purification parameters are summarized in Table 1. These purified proteins were analyzed by SDS-PAGE and detected at the expected molecular weight (Figure 6B).

### 2.3. Functional Characterization of Key Proteins in GAG-PUL

#### 2.3.1. RiPL8A and Its Truncated Mutants

**Figure 7:**
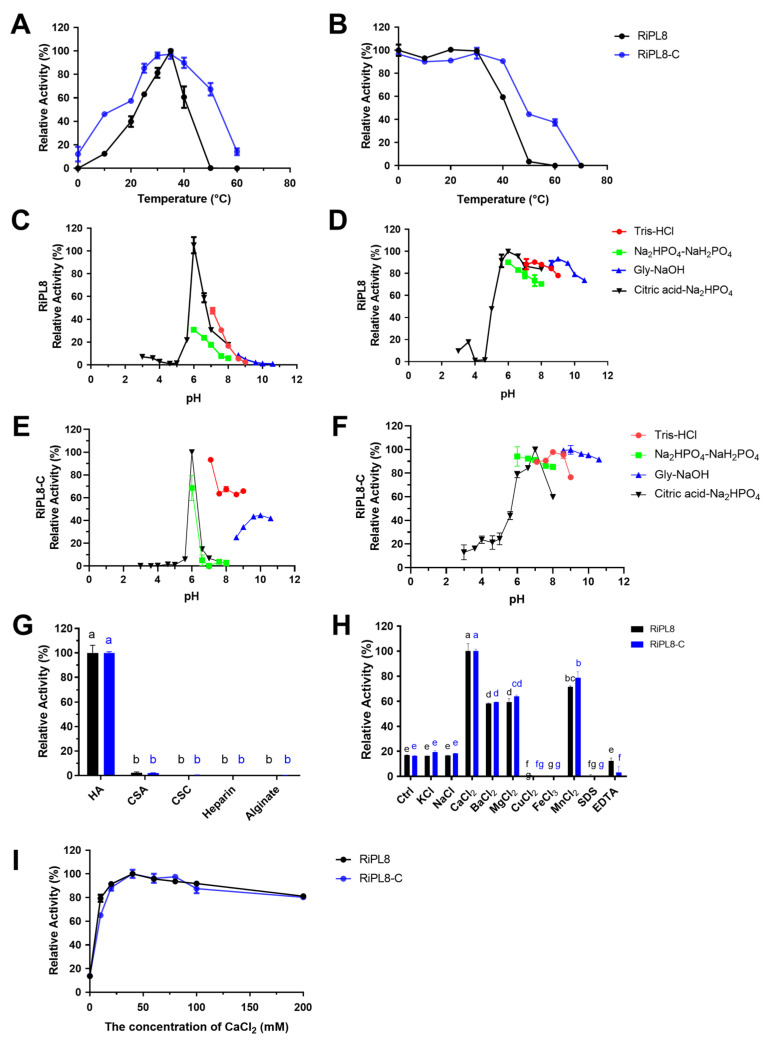
Biochemical characterization of RiPL8 and its truncated mutant. Optimal temperature (**A**), thermal stability (**B**), optimal pH ((**C**), RiPL8; (**E**), RiPL8-C), pH stability ((**D**), RiPL8; (**F**), RiPL8-C), substrate specificity (**G**), effect of metal ions (**H**), and effect of CaCl_2_ concentration (**I**) of RiPL8 and RiPL8-C. Values represent the mean of three replicates ± standard deviation, and different letters indicate significant differences at *p* < 0.05 between groups.

The optimal temperature (T_opt_) of RiPL8 and RiPL8-C was both 35 °C, while RiPL8 retained 59.35 ± 2.29% of its activity but RiPL8-C retained 90.52 ± 0.55% after 1 h of pre-incubation at 40 °C (Figure 7A,B). In addition, the optimal pH for RiPL8 and RiPL8-C was found to be 6.0 in Na_2_HPO_4_–citric acid buffer (Figure 7C,E), and they remained steady in a pH range between 5.6 and 10.6 in different buffers (Figure 7D,F). In order to ascertain substrate spectrum, RiPL8 and RiPL8-C were assayed against different substrates, including HA, CSA, CSC, heparin, and alginate. The results indicate that RiPL8 was an HA-exclusive lyase, consistent with RiPL8-C (Figure 7G). Figure 7H depicts the effects of metal ions, surfactants, or chelators on enzymatic activities. The catalytic capability of RiPL8 and RiPL8-C was significantly increased by Ca^2+^, Ba^2+^, Mg^2+^, and Mn^2+^. As shown in Figure 7I, both enzymes displayed optimal activity (≥80%) at 10–200 mM CaCl_2_.

**Figure 8:**
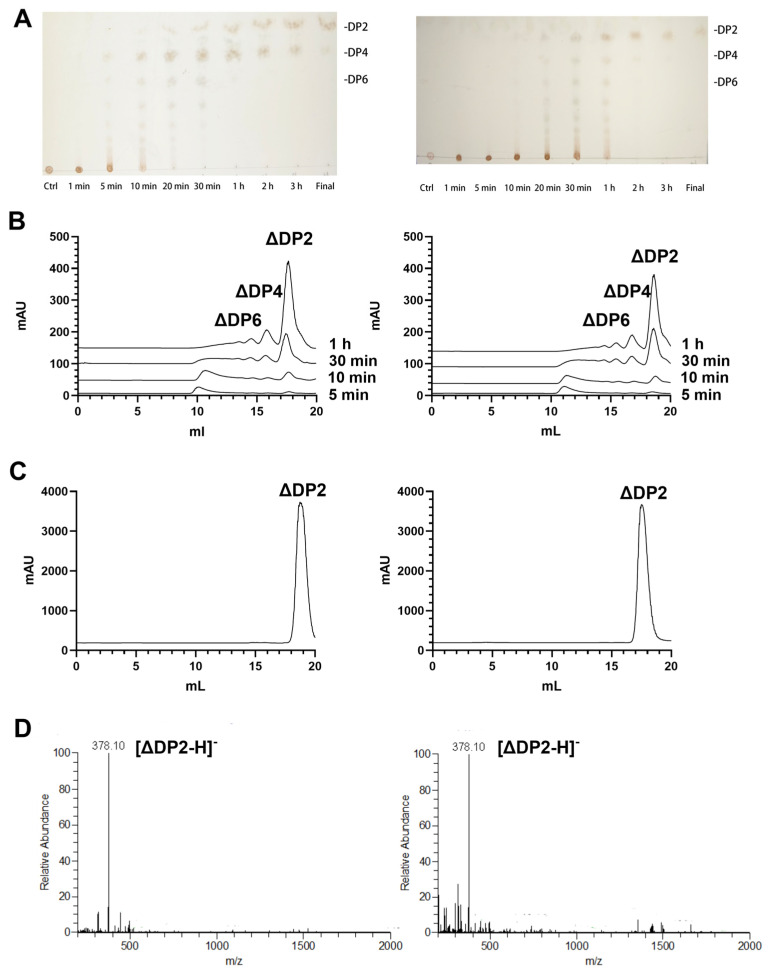
Degradation mode and the end products of RiPL8 and its truncated mutant. (**A**,**B**) The time course of HA degradation by RiPL8 (**left**) and RiPL8-C (**right**) using TLC (**A**) and size exclusion chromatography (**B**). (**C**,**D**) Analysis of the end products of RiPL8 (**left**) and RiPL8-C (**right**) using size exclusion chromatography (**C**) and ESI-MS analysis (**D**).

The degradation mechanisms of RiPL8 and RiPL8-C were evaluated using TLC and size exclusion chromatography analysis (Figure 8A,B). A small number of low-molecular-mass oligosaccharides were detectable during the initial degradation phase. With the continuous degradation, the oligosaccharides in the intermediate fragment gradually become the single end-product (Figure 8C). Then, these end-products’ peak were collected, and the unsaturated HA disaccharides as the final products of RiPL8 and RiPL8-C were both identified by ESI-MS (Figure 8D). Therefore, the action mode of RiPL8 was not changed by truncating the mutant.

The specific activities of RiPL8 and RiPL8-C assessed were 2.06 U/mg and 5.22 U/mg, respectively (Table 1). The enzyme kinetics of RiPL8 and its truncated mutant towards HA are summarized in Table 2. The *K*_m_ and *k*_cat_/*K*_m_ values of RiPL8 were similar to that of RiPL8-C. Thus, the catalytic activity and binding ability of RiPL8 were not significantly altered by the truncating mutant.

**Figure 9:**
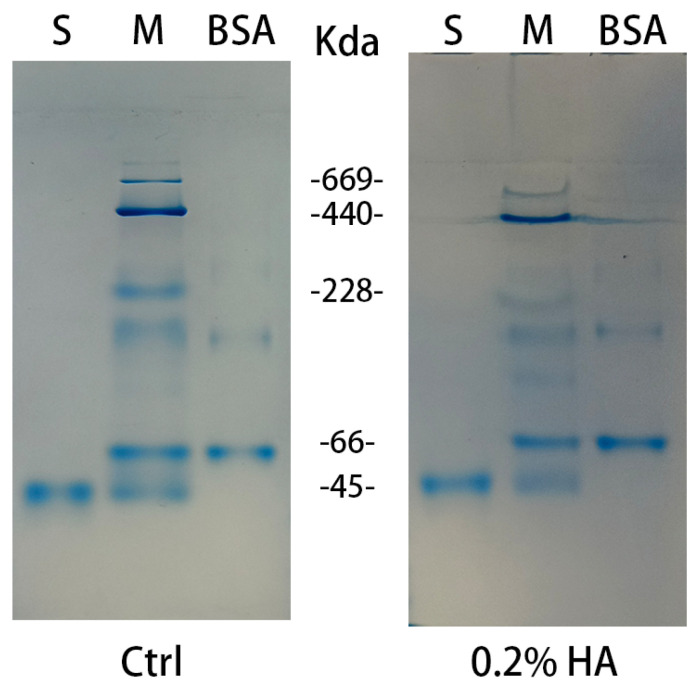
The HA binding ability of RiPL8-N. Affinity non-denaturating electrophoresis was performed in 8% native PAGE gels by preparing with or without HA. Lane S, RiPL8-N; Lane M, native-molecular-weight markers.

Likewise, the function of the N-terminal domain of RiPL8 was investigated. RiPL8-N was catalytically inactive by incubation with different substrates. Additionally, the binding of RiPL8-N to HA was analyzed using native PAGE with or without polysaccharides embedded in the gel (Figure 9). The mobility of RiPL8-N in native PAGE with HA coincides with that without HA. The above result revealed that RiPL8-N did not possess the ability to catalyze and bind HA.

#### 2.3.2. RiPL35 and RiPL38

**Figure 10:**
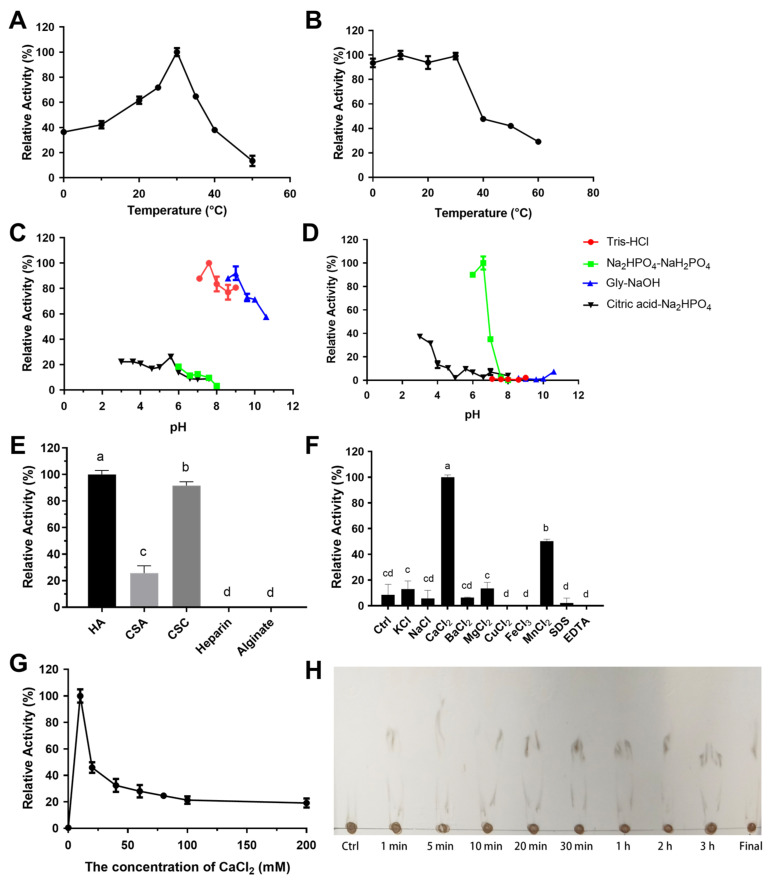
Biochemical characterization of RiPL35. Optimal temperature (**A**), thermal stability (**B**), optimal pH (**C**), pH stability (**D**), substrate specificity (**E**), effect of metal ions (**F**) and effect of CaCl_2_ concentration (**G**) and the time course of HA degradation using TLC (**H**). Values represent the mean of three replicates ± standard deviation, and different letters indicate significant differences at *p* < 0.05 between groups.

The Topt for RiPL35 was 30 °C (Figure 10A), while the enzyme retained ≥90% relative activity after one hour pre-incubation at 0 to 30 °C (Figure 10B). According to Figure 10C,D, RiPL35 showed a pH optimum of 7.6 in Tris-HCl buffer, and stability was optimum at pH 10.0 to 10.6. The spectrum of the substrate is displayed in Figure 10E. RiPL35 exhibited a degradative activity only for HA and CS, with relative activity values of 100.00 ± 3.08% (HA), 25.72 ± 0.06% (CSA), and 91.63 ± 2.88% (CSC). The effects of metal ions, detergents, or chelators on RiPL35 activity are presented in Figure 10F. Ca^2+^ ions significantly increased RiPL35 activity (more than 10 times). As shown in Figure 10G, the activity of RiPL35 was highly sensitive to the Ca^2+^ ion concentration. It was obvious that the activity reached its highest at 10 mM CaCl_2_ and decreased rapidly.

**Figure 11:**
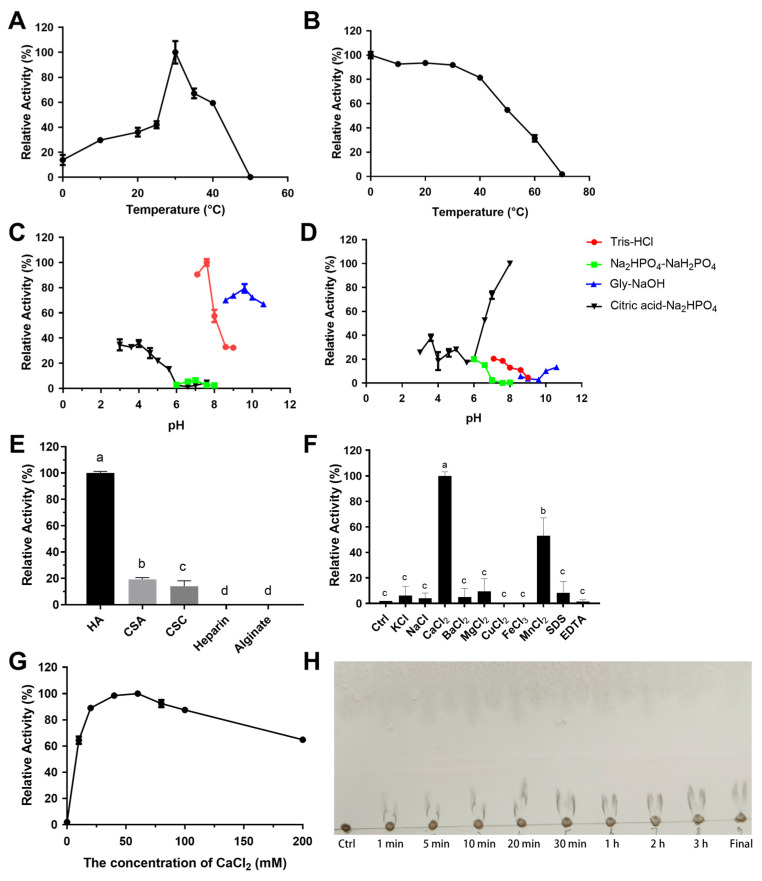
Biochemical characterization of RiPL38. Optimal temperature (**A**), thermal stability (**B**), optimal pH (**C**), pH stability (**D**), substrate specificity (**E**), effect of metal ions (**F**) and effect of CaCl_2_ concentration (**G**) and the time course of HA degradation using TLC (**H**). Values represent the mean of three replicates ± standard deviation, and different letters indicate significant differences at *p* < 0.05 between groups.

In addition, the Topt and optimal pH of RiPL38 were 30 °C and 7.6, respectively (Figure 11A,C). The stability of RiPL38 was retained over 50% of its peak activity when incubated at 0–50 °C for 1 h or within a pH range of 6.6–8.0 for 12 h (Figure 11B,D). Regarding substrate specificity, RiPL38 demonstrated high substrate specificity for HA (100.00 ± 1.25%) and low activity for CSA (19.37 ± 1.16%) and CSC (14.04 ± 4.11%) (Figure 11E). The activity of RiPL38 was significantly increased by Ca^2+^ and Mn^2+^ (Figure 11F). Further investigation demonstrated that RiPL38 maintained more than 60% activity at 0–200 mM CaCl_2_ and showed the highest activity at 60 mM (Figure 11G).

Similarly, the degradation mechanisms and final products of RiPL35 and RiPL38 have also been investigated. The low enzymatic activity of RiPL35 and RiPL38 limited TLC analysis to demonstrating their HA-degrading capability, while their distinct modes of action and final degradation products remained challenging to determine unambiguously (Figure 10H).

## 3. Discussion

*Roseihalotalea indica* gen. nov. sp. nov. TK19036^T^, a novel marine *Bacteroidetes*, was previously isolated from the southwest Indian Ocean [18]. Genomic analysis revealed a diverse array of CAZymes and PULs within its genome, capable of targeting various substrates including alginate, arabinan, arabinoxylan, fructan, and pectin [17]. In this research, we observed that *Roseihalotalea indica* gen. nov. sp. nov. TK19036^T^ can only utilize CS by the sole carbon source cultivation. To further identify the CAZymes and PUL of GAG degradation, we performed CAZyme gene cluster analysis via CGC finder in the dbCAN3 meta server [19], but could not obtain further annotation.

Multi-omics technology has been developing rapidly with technological advances, providing new methods for the discovery and mining of enzymes from the microbiome [20]. The combined approach of sequence similarity network (SSN) + proteomics has been applied successfully to discover and characterize the isethionate sulfolyase from *Bilophila wadsworthia* [21,22]. Drawing from this approach, an upregulated gene cluster was identified via DIA quantitative proteomics. The PUL harbors a pair of genes with significant homology to the susC and susD genes of the starch utilization system (Sus) in *B. thetaiotaomicron* [23], three genes for polysaccharide lyases (PL), one gene for carbohydrate esterase (CE), and a string of four genes annotated as hypothetical proteins. Besides the four hypothetical proteins, the expression of the remaining six genes was significantly upregulated. Thus, we honed in on the enzymatic properties of each CAZyme in this PUL. The three PLs (RiPL8, RiPL35, and RiPL38) were all successfully expressed in the recombinant strain, *E. coli*/pET28a(+), but RiCE19 was not expressed in soluble form after optimization. RiPL8, RiPL35, and RiPL38 were therefore further characterized.

Based on the CAZy database, GAG-lyase can be grouped into 14 PL families as follows: PL-6, -8, -12, -13, -15, -16, -17, -21, -23, -29, -30, -33, -35, and -37. Compared to members of other PL families, the PL-8 family is the most well-studied GAG-lyase, but certain members also exhibit alginate lyase activity. RiPL8, unlike the PL-8 family members, consists of two domains: a N-terminal “Alginate_lyase” module and a C-terminal “GAG_Lyase super family” domain, but it only showed degradation activity toward HA. Using truncation mutants, the C-terminal “GAG_Lyase super family” domain was determined as the catalytic domain of RiPL8. Surprisingly, the N-terminal “Alginate_lyase” module was seemingly devoid of function, which affected neither the HA binding nor the catalytic activity of RiPL8. The exact function will require further study.

The PL35 family has been exhaustively studied by Wei et al. in recent years. Eight PL35 GAG-lyases from diverse microbial species were characterized; it was found that not only can all degrade three types of GAGs—HA, CS, and HS—but also one of them can even degrade alginate [24]. RiPL35 exhibited the ability to degrade not only HA, but also CS, despite weak activity to CSA. Meanwhile, the activity of RiPL35 was extremely sensitive to increased Ca^2+^ levels. When the Ca^2+^ concentration was 10 mM, the enzymatic activity reached the highest level, and decreased quickly in.

At present, four members of the PL-38 family have been identified and characterized. There are two alginate lyases, Aly38A [25] from *Agarivorans* sp. B2Z047 and BoPL38 [26] from *Bacteroides ovatus*, and two β-1,4-glucuronan lyases, CUL-I [27] from *Brevundimonas* sp. SH203 and TpPL38A [28] from *Trichoderma parareesei*. RiPL38 is the first reported PL38 family member with the degradation activity of GAGs, exhibiting high activity towards HA but weak activity against CS. These data broadened the substrate spectrums of PL38. However, we were not able to obtain the degradation mechanisms and end products of RiPL35 and RiPL38, due to low activity.

Unexpectedly, the three PLs all showed the highest activity on HA compared to other substrates. This result was not in accordance with the findings of the sole carbon source cultivation. Therefore, the reason for this phenomenon requires further investigation.

## 4. Materials and Methods

### 4.1. Materials and Strains

Glycosaminoglycans (GAGs), including sodium hyaluronate, chondroitin sulfate sodium, and heparin sodium, were purchased from Macklin (Macklin Inc., Shanghai, China). *Roseihalotalea indica* gen. nov. sp. nov. TK19036^T^, generously provided by Prof. Kai Tang, was cultured in 2216E medium (Qingdao Haibo Biotech Co., Ltd., Qingdao, China). *E. coli* BL21 (DE3) (TaKaRa, Dalian, China) served as the host for recombinant protein expression in LB medium with 30 mg/mL of kanamycin.

### 4.2. Prediction of GAG-PUL in Roseihalotalea indica gen. nov. sp. nov. TK19036^T^

The genome assemblies of *Roseihalotalea indica* gen. nov. sp. nov. TK19036^T^ (GenBank: CP120682) were deposited in GenBank in previous studies [17,18]. The Rapid Annotation using Subsystem Technology (RAST) (https://rast.nmpdr.org/, accessed on 28 March 2025) was used to annotate the genome. The dbCAN3 meta server (http://bcb.unl.edu/dbCAN2/, accessed on 27 March 2025) identified potential carbohydrate-active enzyme (CAZyme) genes, and their functions were accurately predicted using the CAZy database families. The BLASTp was used to search proteins and determine their sequence similarity. The CAZyme gene cluster (CGC) Finder in the dbCAN3 database was used for carbohydrate-active enzyme cluster annotation.

### 4.3. Sole-Carbon-Source Cultivation of Roseihalotalea indica gen. nov. sp. nov. TK19036^T^

The sole-carbon-source cultivation was performed as previously described [29]. Briefly, cultivation experiments used a sole-carbon-source medium designed based on 2216E medium as the background, where organic carbon and nitrogen components were removed and an appropriate amount of NH_4_Cl was added, with 0.3% (*w*/*v*) GAGs (hyaluronate, chondroitin sulfate, and heparin) serving as the sole carbon sources and glucose as the control group. Cells in the logarithmic growth phase were collected by centrifugation at 1000 rpm for 30 min at 4 °C and subsequently washed three times with sterile artificial seawater, prior to inoculation. The cells were cultured in a medium containing a single carbon source, in triplicate, at a temperature of 25 °C for a duration of 10 days, with glucose serving as the control condition. The growth curve was established by measuring OD_600_.

### 4.4. DIA Quantitative Proteomics Analysis of Roseihalotalea indica gen. nov. sp. nov. TK19036^T^

The precipitate was dissolved in a protein lysate solution containing 8 M urea, 1% SDS, and a protease inhibitor cocktail. After sonication on ice for 2 min and centrifugation at 12,000× *g* for 20 min at 4 °C, the protein content of 1 μL of the supernatant was determined using the ProteoAnalyzer (M5350AA). DIA quantitative proteomics analysis was conducted at Shanghai Majorbio Bio-Pharm Technology Co., Ltd. (Shanghai, China), and the data were analyzed on the online Majorbio Cloud Platform (https://cloud.majorbio.com, accessed on 16 May 2025).

Spectronaut software (Version 19) was used to search the DIA raw data. The parameters are as follows: the peptide length range was set to 7–52; the enzyme cutting site was trypsin/P; the maximum missed cleavage site was 2; the carbamidomethylation of cysteines was a fixed modification; the oxidation of methionines and protein N-terminal acetylation were variable modifications; Protein FDR ≤ 0.01; Peptide FDR ≤ 0.01; Peptide Confidence ≥ 99%; and XIC width ≤ 75 ppm. The protein quantification method was MaxLFQ. *p*-values and fold change (FC) for the proteins between the two groups were calculated using R package “*t*-test”. The thresholds of fold change (>1.2 or <0.83) and *p*-value < 0.05 were used to identify differentially expressed proteins (DEPs).

### 4.5. Sequence Analysis of GAG-Lyases-Encoding Gene

SignalP 6.0 (http://www.cbs.dtu.dk/services/SignalP-6.0/, accessed on 8 May 2025) was employed to determine the presence of the signal peptide. PSORTb v 3.0.3 (https://www.psort.org/psortb/, accessed on 8 May 2025) was used to predict protein subcellular localization. The ExPASy ProtParam tool (https://web.expasy.org/compute_pi/, accessed on 8 May 2025) was utilized to estimate the molecular weight (Mw) and theoretical isoelectric point (pI) of the target proteins. Analysis of the conserved domain architecture was performed using the NCBI Conserved Domain Database and the Conserved Domain Architecture Retrieval Tool (CDART). Evolutionary analysis was conducted employing the maximum likelihood method with 1000 bootstrap replicates (MEGA, version 11).

### 4.6. Cloning, Expression, and Purification of GAG-Lyases

The genome sequence of *Roseihalotalea indica* gen. nov. sp. nov. TK19036^T^ served as the foundation for the design of the specific primers incorporating *Nde I*/*Xho I* restriction sites, as detailed in Table 3. The amplification products were inserted into the pET-28a(+) plasmid to construct over-expression vectors.

After amplification of recombinant plasmids by transformation into the *E. coli* BL21 (DE3) cells, they were cultured on LB medium with kanamycin. Subsequently, when cells reached OD_600_ = 0.5, 0.02 mM IPTG was added to induce protein overexpression at 18 °C for 24 h. After breaking the cells by ultrasonic treatment, the supernatant containing recombinant proteins was collected by centrifugation. The recombinant proteins were purified in a single step via Ni-affinity chromatography utilizing the HisTrap™ HP column (Cytiva, Uppsala, Sweden), and the purity and molecular weight of the purified protein were verified by SDS-PAGE. The concentrations of the purified proteins were quantified using the BCA (bicinchoninic acid) protein estimation kit (New Cell & Molecular Biotech, Suzhou, China).

### 4.7. Enzymatic Activity Assay of GAG-Lyases

The enzymatic activity assay was conducted by incubating serially diluted recombinant proteins (100 µL) with a 0.2% (*w*/*v*) standard hyaluronate substrate (900 µL) in a 20 mM Tris buffer containing 10 mM CaCl_2_ at pH 7.0. The reaction mixtures were maintained at 35 °C for 10 min. Subsequently, the 4,5-unsaturated products were quantified using a UV spectrophotometer UH5300 (Hitachi, Tokyo, Japan) at a wavelength of 232 nm. Enzymatic activity was determined by measuring the increase in absorbance (ΔA232) and applying a molar extinction coefficient of 5500 M^−1^ cm^−1^ for unsaturated glucuronate residues. One unit of lyase activity (U) was defined as the amount of enzyme required to produce one micromole of unsaturated oligosaccharides per minute under the specified conditions.

This study examined the kinetic parameters of RiPL8 and the truncated mutant across the concentrations of HA from 0.05% to 1.60% (*w*/*v*). The Michaelis–Menten kinetic model was applied to calculate these kinetic parameters, with nonlinear regression curves generated by GraphPad Prism 8.0.0 (San Diego, CA, USA). Enzymatic assays were performed in biological triplicates, with specific activity values reported as mean ± standard deviation (SD).

### 4.8. Biochemical Characterization of GAG-Lyases

The impact of pH on enzymatic activities was examined using a variety of buffer systems, including 20 mM glycine-NaOH (pH 8.6–10.6), 20 mM Tris-HCl (pH 7.05–8.95), 20 mM Na_2_HPO_4_-NaH_2_PO_4_ (pH 6.0–8.0) and 20 mM Na_2_HPO_4_-citric acid (pH 3.0–8.0). The pH stability of the enzymes was checked in different solvents for 12 h of incubation at 4 °C. Additionally, the degradation system was analyzed across a temperature range from 0 °C to 60 °C to determine the optimum catalytic temperature. The thermostability was estimated by pre-incubating the enzyme in a Tris buffer (20 mM, pH 7.3) at the above temperatures (0–60 °C) for a period of 1 h and then comparing with the control without incubation. The influence of Ca^2+^ ions was evaluated by assessing enzyme activities in a Tris buffer (pH 7.0) containing varying concentrations of CaCl_2_ (0–200 mM) at the optimal temperature and pH. Relative enzymatic activity was calculated by comparing it to the maximum enzymatic activity, which was set as 100% in these experiments. All assays were performed in triplicate, and enzymatic activity was reported as the mean ± standard deviation (SD).

### 4.9. Analysis of Reaction Pattern and End Products of GAG-Lyases

To elucidate the mode of action, TLC (Thin-layer chromatography) and AKTA Purifier systems were utilized. In summary, the digests of 0.20% (*w*/*v*) HA by the enzymes (0.02 U/mL) were traced at each optimal condition. Aliquots (200  μL) of the reactants were removed at different time points and analyzed using silica gel 60 F254 TLC plates (Merck Millipore, Darmstadt, Germany) with the mobile phase consisting of n-butanol, formic acid, and water in a 2:2:1 (*v*/*v*/*v*) ratio. The plates were stained with diphenylamine-aniline-phosphate (DPA) reagent and subsequently charred at 130 °C to visualize carbohydrate bands. In addition, aliquots were analyzed through size exclusion chromatography on a Superdex 30 Increase 10/300 GL column (Cytiva, Chicago, MA, USA) eluted with 0.20 M NH_4_HCO_3_ at a flow rate of 0.8 mL/min and monitored at 232 nm using a UV detector. The final products were desalted and further characterized via negative-ion ESI-MS.

## 5. Conclusions

In this study, the GAG-PUL of the novel marine *Bacteroidetes*, *Roseihalotalea indica* gen. nov. sp. nov. TK19036^T^, was identified through sole carbon source cultivation and DIA quantitative proteomics. Three PLs from the GAG-PUL, RiPL8, RiPL35, and RiPL38, were characterized in terms of their enzymatic properties. RiPL8 has an unknown N-terminal domain and a catalytic C-terminal domain that specifically degrades HA. The enzymatic activity of RiPL35 is influenced by Ca^2+^ ion concentration, with an optimal activity observed at 10 mM. RiPL38 is the first PL38 family member known to degrade HA and CS. Collectively, our study uncovers that *Roseihalotalea indica* gen. nov. sp. nov. TK19036^T^ harbors an unexpected GAG degradation gene cluster, with the encoded GAG-lyases exhibiting substrate specificities distinct from those of the host organism.

## Figures and Tables

**Table 1:** Purification parameters of recombinant proteins.

Protein	Sample	Total Protein (mg)	Total Activity (U)	Specific Activity (U/mg)	Fold	Recovery (%)
RiPL8	Crude enzyme	212.04	59.49	0.28	1	100
	Affinity chromatography	16.07	33.13	2.06	7.33	55.70
RiPL8-C	Crude enzyme	151.97	336.94	2.22	1	100
	Affinity chromatography	16.65	86.88	5.22	2.36	25.79
RiPL35	Crude enzyme	229.73	9.10	0.04	1	100
	Affinity chromatography	31.00	3.76	0.12	3.10	41.29
RiPL38	Crude enzyme	239.80	6.58	0.03	1	100
	Affinity chromatography	38.16	4.06	0.11	3.93	61.69

**Table 2:** Specific activities of full-length RiPL8 and its truncated mutants.

Protein	*V*_max_ (nmol·s^−1^)	*K*_m_ (mM)	*k*_cat_ (s^−1^)	*k*_cat_/*K*_m_ (mM·s^−1^)
RiPL8	3.69	0.38 ± 0.12	32.59 ± 3.80	84.85
RiPL8-C	0.66	0.23 ± 0.09	9.09 ± 1.10	38.73

**Table 3:** Primers used in this study.

Primers	Sequence (5′ to 3′)	Usage
RiPL8-F	gtgccgcgcggcagccatatgTACGAACATCCCGGTGGCA	Expression of RiPL8 and RiPL8-N
RiPL8-R	gtggtggtggtggtgctcgagCTATATCTTGTTGGGGGTTTGGA	Expression of RiPL8 and RiPL8-C
RiPL8-N-F	gtgccgcgcggcagccatatgCCCCTCAGAAACTACGGCG	Expression of RiPL8-N
RiPL8-C-R	gtggtggtggtggtgctcgagGTCGCCGTAGTTTCTGAGGGG	Expression of RiPL8-C
RiPL35-F	gtgccgcgcggcagccatatgATGGCAAGGGCCAGCACT	Expression of RiPL35
RiPL35-R	gtggtggtggtggtgctcgagATTTTCTGCGGTTAGGCGC	Expression of RiPL35
RiPL38-F	gtgccgcgcggcagccatatgCAGCCGTCGCCTCTGACC	Expression of RiPL38
RiPL38-R	gtggtggtggtggtgctcgagCGGGACTTTCTTTGCTTCGG	Expression of RiPL38

## Data Availability

The data presented in this study are available on request from the corresponding author.
